# Editorial: Cellular and molecular factors that drive the behavior of oligodendrocyte progenitor cells in physiological conditions and disease

**DOI:** 10.3389/fncel.2023.1145627

**Published:** 2023-02-06

**Authors:** Davide Lecca, Wia Baron, Arthur M. Butt

**Affiliations:** ^1^Department of Pharmaceutical Sciences, Università degli Studi di Milano, Milan, Italy; ^2^Department of Biomedical Sciences of Cells and Systems, University of Groningen, University Medical Center Groningen, Groningen, Netherlands; ^3^School of Pharmacy and Biomedical Science, University of Portsmouth, Portsmouth, United Kingdom

**Keywords:** oligodendrocyte progenitor cell (OPC), remyelination failure, inflammation, myelin, extracellular environment, energy metabolism

Oligodendrocyte progenitor cells (OPCs) are a subtype of glia giving rise to the myelin producing cells in the central nervous system. OPCs are not passive actors unavoidably committed to become oligodendrocytes (OLs) through a precise intrinsic program. As highlighted in this Research Topic, OPCs are extremely plastic and functionally heterogeneous and adapt their program in response to a multitude of signals both in physiological and pathological conditions (Marisca et al., 2020; Boda et al., 2022).

Local environmental cues drive OPC diversity during embryogenesis, development, and adulthood. Green et al. demonstrate that, in zebrafish, peripheral sensory neurons at the dorsal root entry zone are responsible for the development of a very specific subset of OL lineage cells. These sensory OL lineage cells lack *mbpa* transcripts, wrap around axons, affect the function of peripheral sensory nerves, but behave differently than typical oligodendrocytes.

Fekete and Nishiyama provide an overview of environmental signals that direct OPC fate, differentiation, maturation, and myelination and discuss in detail molecular mechanisms of how neuronal-derived BDNF and L1CAM that share common signaling pathways, control OPC differentiation. Differentiation and myelination are complex physiological processes that take place simultaneously in the same areas, and a relevant set of extrinsic factors often trigger different cellular programs thereby selectively acting in the correct subset of cells. Elucidating how extracellular signals are integrated in OPCs would provide new insights in the pathophysiology of these cells and is a therapeutic goal in regenerative medicine. For example, sonic hedgehog (Shh) has a plethora of effects that regulate both OPC proliferation and differentiation (Ruat et al., 2015). In the canonical Shh signaling, its key effector Smo triggers a downstream cascade through the transcription factor Gli1. Shh/Smo signaling was found to be reactivated in demyelinating lesions during remyelination (Ferent et al., 2013). Clobetasol, an FDA-approved promyelinating drug, modulate Smo in oligodendroglial cells and promote a Gli2-dependent modulatory pathway. For this reason, the identification of novel Smo agonists is a therapeutic goal in regenerative medicine. Del Giovane et al. investigated the activity of the recently developed Smo-binding compound GSA-10. *In vitro*, GSA-10 treatment increased axonal ensheathment and myelin compaction more efficiently than Clobetasol. Moreover, in mice subjected to lysolecithin-induced demyelination, GSA-10 promoted recruitment and differentiation of OPCs into lesions, supporting its role as a pro-myelinating drug.

Successful differentiation of resident OPCs is a key event to restore damaged circuitry and myelin loss that takes place during chronic inflammation. However, a non-permissive environment negatively affects OPC maturation capabilities (Coppolino et al., 2018). Göttle et al. focused on the endogenous envelope protein (ENV), a negative regulator of OPC maturation acting at the TLR-4 receptor. They investigated how ENV could be neutralized to improve remyelination and identified pharmacological agents acting at TLR-4 that restore oligodendroglial differentiation and fully rescue myelination deficits by counteracting ENV-dependent changes in mitochondrial integrity.

During differentiation, OPCs have to fully rewire their energy metabolism, to support axons in response to environmental cues (Marangon et al., 2022). It is crucial to understand how certain inputs can regulate OL metabolism, and how alterations in cell-to-cell communication can be at the basis of neurodegeneration or remyelination failure. In their review, Narine and Colognato describe OL metabolism and the signaling events that regulate the key steps of differentiation, maturation, and myelination. Extrinsic factors such as drugs and diet can disrupt OL energy metabolism thus altering the homeostasis of the CNS and contributing to the development of neurodegenerative diseases. Along these lines, targeting OL bioenergetic pathways represent a new opportunity to boost their regenerative potential.

The mitochondrial aspartate-glutamate transporter AGC1 is an essential player for correct glucose oxidation. Downregulation of AGC1 in OPCs inhibits proliferation and leads to precocious differentiation, together with a reduction in the levels of *N*-acetyl-aspartate, an important acetate donor for myelin lipid synthesis, and a potential source of acetate in histone acetylation (Profilo et al., 2017). Poeta et al. show that down-regulation of AGC1 affected the balance between acetylation and deacetylation in OPCs. This epigenetic defect could be part of the pathogenetic mechanism underlying AGC1 deficiency, an ultrarare genetic disease characterized by global hypomyelination and brain atrophy.

Epigenetic regulation functionally governs differentiation of OPCs. The epigenetic enzyme PRMT5 is a protein arginine methyl transferase responsible for the symmetric methylation of arginine residues on histone tails. In OPCs, PMRT5 is predominantly localized in the cytosol, where it methylates several RNA-binding proteins involved in RNA processing. Using a mass spectrometry-based proteomic approach and iTRAQ labeling of nuclear and cytosolic protein extracts, Dansu et al. identify new direct interactors of PRMT5 in OPCs and show its potential role in biological processes such as RNA stability, transcription, and migration. HMGB1, the most abundant nuclear non-histone binding protein associated to chromatin, is secreted during cellular stress and senescence, and induces inflammation. Rouillard et al. demonstrate that HMGB1 arrests OPC differentiation through a TLR2-mediated mechanism and contributes to remyelination failure in a model of lysolecithin-induced demyelination. Interestingly, high levels of HMGB1 released by senescent neural precursor cells were previously described within human demyelinated multiple sclerosis lesions (Nicaise et al., 2019), thus suggesting the clinical relevance of this inhibitory factor in pathological conditions.

Considerable myelin loss and consequent progressive cognitive deficit were also found in aging brain (Rivera et al., 2021). Rivera et al. describe the molecular consequences of aging in OPCs and focus on the epidermal growth factor (EGF). Network analysis on the aging myelinating OL transcriptome revealed core genes belonging to the EGF signaling commonly dysregulated in Alzheimer's disease and multiple sclerosis. The identification of drugs that target this signaling pathway could positively influence rejuvenation and myelin regeneration.

The cellular and molecular context makes OPCs highly heterogeneous and plastic both in time and space with mechanisms largely unknown. The articles in this Research Topic contributed to gain insights in the multitude of signals received by OPCs and how these signals are processed to determine OPC behavior. Many, if not all, neurodegenerative diseases are likely to share common pathogenetic mechanisms; deciphering how OPCs rewire their program in response to injury signals may open new therapeutic chances to facilitate CNS homeostasis in disease.

## Author contributions

DL wrote the manuscript. AB and WB revised the manuscript. All authors have read, revised, and approved the submitted version.
